# Beliefs about Lying and Spreading of Dishonesty: Undetected Lies and Their Constructive and Destructive Social Dynamics in Dice Experiments

**DOI:** 10.1371/journal.pone.0077878

**Published:** 2013-11-13

**Authors:** Heiko Rauhut

**Affiliations:** 1 Institute of Sociology, University of Zurich, Zurich, Switzerland; 2 Department of Humanities, Social and Political Sciences, ETH Zurich, Swiss Federal Institute of Technology, Zurich, Switzerland; University of Maribor, Slovenia

## Abstract

Field experiments have shown that observing other people littering, stealing or lying can trigger own misconduct, leading to a decay of social order. However, a large extent of norm violations goes undetected. Hence, the direction of the dynamics crucially depends on actors’ beliefs regarding undetected transgressions. Because undetected transgressions are hardly measureable in the field, a laboratory experiment was developed, where the complete prevalence of norm violations, subjective beliefs about them, and their behavioral dynamics is measurable. In the experiment, subjects could lie about their monetary payoffs, estimate the extent of liars in their group and make subsequent lies contingent on information about other people’s lies. Results show that informed people who underestimate others’ lying increase own lying more than twice and those who overestimate, decrease it by more than half compared to people without information about others’ lies. This substantial interaction puts previous results into perspective, showing that information about others’ transgressions can trigger dynamics in both directions: the spreading of normative decay and restoring of norm adherence.

## Introduction

Publicly visible norm violations may subsequently trigger more norm violations and eventually set off dynamics of normative decay and disorder. This dynamics has recently been tested in a series of field experiments where graffiti, litter, unreturned shopping carts, and illegal parking caused people to violate the same and even other norms [1]. Similarly, it has been shown that people litter if they observe others littering [2], and people lie more if they observe others lying [3], [4]. The contagiousness of disorder tends to be particularly strong if there is nobody around giving cues that show respect for social order [5].

This dynamics may be explained by a mechanism linking the perceived prevalence of a certain behavior with subjective beliefs about its common approval. People hold beliefs about the average behavior, i.e., about the “descriptive norm”, and make inferences about its appropriateness, i.e., about the “injunctive norm” [6]. In this way, occurrences of public norm violations may make people aware of a larger than initially believed prevalence of the behavior, trigger reassessments of its common approval and result in an amplification of disorder and normative decay.

However, many norm violations are not publicly visible but conducted in private. Two-timing, tax evasion, consumption of pornography, visits to prostitutes or alcohol abuse are only some of many examples where norm violations are typically concealed from others; consequently, large parts remain in the dark. Therefore, the complete rate of norm violations consists of detected and undetected norm violations. Hence, normative dynamics and normative decay are crucially dependent on actors’ subjective beliefs about the rate of undetected norm violations. If actors perceive others’ norm violations, their beliefs about the additional extent of undetected norm violations are crucial for their evaluation of the appropriateness of the behavior and their own decision to adhere or violate the norm.

If actors underestimate the complete extent of norm violations, the proposed dynamics of the above mentioned authors hold if certain conditions are met [7]. Those underestimating others’ transgressions may perceive occurrences of others’ norm violations as relatively frequent or strong if they are informed about the true extent of norm violations. As a consequence, they increase their subjective estimates about the extent of norm violations and subsequently perform more own norm violations. In a classic paper [8] sociologist Heinrich Popitz already outlined this idea of a “preventive effect of ignorance”. That means that an actor’s ignorance of other peoples’ norm violations has a deterrent effect on his or her norm-related behavior. Lifting the ‘veil of ignorance’ is expected to increase the extent of norm violations. An example is the Kinsey report [9] on sexual behavior. The publication of the report at that time had the consequence of changing sexual behaviors and norms of sexual conduct [4], [7], [10]. Of course, information on the true amount of norm violations does not always lead to an upward spiral of transgressions [7]. First, and almost trivially, potential transgressors do not always gain from norm violations. Secondly, the disclosure of norm violations is often paralleled by increasing sanctions, stigmatization and strengthening of the legitimation of the norm as in the case of child abuse by catholic priests [7]. Hence, adjustment of an actor’s underestimation of transgressions does not necessarily lead to an increase in his or her own propensity to violate the norm.

So far we have assumed that actors underestimate the amount of other peoples’ transgressions. However, what will happen to those who overestimate from the start? Will the dynamics be inverted? Will those who believe more transgressions are being committed than de facto adjust their beliefs and violate fewer norms if they hear about the true state of the world? If this consequence were true, the dynamics will crucially depend on actors’ subjective beliefs. My research question therefore asks whether information about others’ norm violations will increase transgressions in societies consisting predominantly of believers in too few transgressions and decreasing ones in those with believers in too many. The conjecture that beliefs about the underestimation or overestimation of others’ norm violations are crucial for determining whether the dynamics set off more or fewer norm violations has been raised consecutively in [11], [12], [4], [7].

The conjecture about the interaction between beliefs and behavior is supported by evidence showing that over- as well as underperformance of norm-relevant behaviors is regarded as deviant. Hence, information about norm violations of others can trigger adjustments in both directions – more or less adherence to social norms. Taking the example of norms of environmental protection, people were informed whether their energy consumption level was above or below the average of their neighborhood. Those above the average reduced their energy consumption; however, those below increased it when there was no special mention of their laudable behavior [11].

Whereas norms of energy consumption are one of the few examples where prevalence is directly measurable, the actual extent of most norm violations is unknown to both the researcher and the people in the field making their choices for pro- or antisocial behaviors. Therefore, I developed an experimental design where the prevalence of norm violations can be observed, the accuracy of beliefs measured and the offsetting dynamics of pro- and antisocial behaviors traced. This allows the understanding of the dynamics between objective information, subjective beliefs and the co-evolution of social norms.

## Experimental Design

The dice experiment [13] was used, as in a previous experiment [4], to study the spread of norm violations. This follow-up study of [4] has been extended by introducing the measurement of beliefs about the extent of others’ norm violations. This experimental setup allowed subjects to violate the honesty norm under highly anonymous conditions. In the experiment, subjects performed multiple dice casts in separate booths where they were isolated from others and unable to be observed (see Fig. S1 for experimental instructions). They entered their cast numbers into a tailor-made graphical computer interface and received Swiss Francs according to the number they reported (Fig. S2). The reported number six was an exception and yielded no earnings. This design put subjects with numbers “six” and numbers lower than “five” into a moral conflict between either adhering to the honesty norm or increasing own payoffs by reporting higher payoffs than they would be entitled to.

This design is an improvement to previous designs in that it avoids deception of subjects. This enhances subjects’ trust in the experimenter and serious completion of the experimental task. A truthful and straightforward description of the experimental protocol ensures that honest reports can be regarded as subjects’ willingness to pay a price for honesty. This is a design improvement compared to experiments where subjects are deceived by predetermined dice cast by manipulated computer programs [14]. If subjects think they are being deceived by the experimenter, they may reciprocate deception and act dishonestly in response to the experimenter’s dishonesty. This would bias the results and render inconclusive results.

The sample consisted of 240 subjects, subdivided into 24 experimental sessions of groups of ten. In each period each subject cast a die twelve times and reported each number. There was one trial period and four periods with payments where one cast was randomly selected for payouts. Subjects were randomly allocated to one of three treatments. In the “info treatment”, subjects were informed about the frequency of each reported payoff (zero, one, two, three, four, and five) in their group. In addition, beliefs were elicited by asking for estimates of these frequencies before information feedback (Fig. S3, S4). Belief accuracy was incentivized with monetary payments for good estimates using a quadratic scoring rule (see Table S1 and Materials and Methods for details).

There were two control treatments. The “control belief” treatment consisted of belief elicitation without information feedback. The “control base” treatment was without belief elicitation and without information feedback. Note that the honesty threshold in this design is such that each number is reported twice and average reported payments are 2.5 Swiss Francs per round. Lying is measureable as upward deviance from this honesty threshold in terms of higher average claimed payments and higher frequencies of high reported dots (i.e., fives).

To the best of my knowledge, this is a novel design to measure subjects’ beliefs about others’ honesty in dice experiments. This improves previous dice designs where the contagiousness of lying was measured without controlling for subjective beliefs about others’ honesty [4]. In [4] it was observed that subjects who were informed about others’ lies increased their own propensity to lie compared to a control condition without information feedback. This main effect could, however, not be separated for those underestimating and those overestimating others’ dishonesty. The novel belief measures allow observing the interaction between information feedback and beliefs and can therefore contribute to the existing literature. In this way the research question whether believers in too little honesty will increase and those in too much decrease their own propensity for honest dice reports can be answered.

The implementation of multiple dice casts per subjects in each round was mainly done for two reasons. The first reason was to improve the measurement of beliefs. The elicitation of beliefs requires subjects to estimate the distribution of dice reports in their group. If there are too few dice casts to be estimated, the distribution becomes very noisy and subjects will not make as much effort in generating good estimates. Hence, elicitation of meaningful beliefs requires robust distributions to be estimated. The repetitions in the form of twelve dice casts per round yield a distribution consisting of twelve casts for each of the nine other group members, totaling in 108 dice casts from other group members per round. Accordingly, the distribution to be estimated by each subject is relatively robust. The second argument for introducing multiple dice casts per round was to ensure robust information feedback. If information feedback is driven by too much random noise, it does not give enough information about the lying behavior of others in the group. Consequently, too few dice casts per round in each group hinder studying time trends in lying over the four rounds of the experiment.

With respect to introducing multiple dice casts per subject, it obviously needs a good compromise between guaranteeing enough robustness for eliciting meaningful beliefs and meaningful information feedback on the one hand and allowing for enough randomness for ensuring anonymity for lying on the other hand. This compromise is critical since to cast high numbers twelve times is far more unlikely than it is to throw a high number once. As a result, the identification of liars on the individual level becomes easier the more repetitions there are. All in all, I believe that the choice of twelve repetitions per round in groups of ten subjects represents a good compromise between robustness and anonymity.

## Results

**Figure 1:**
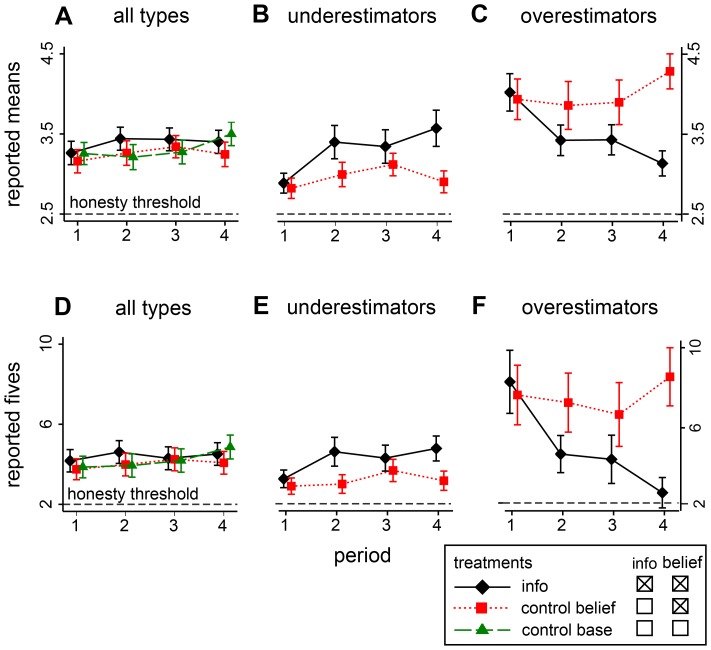
Trend of reported payment claims in means (panels A–C) and fives (panels D–F). Error bars show adjusted 95% confidence intervals such that non-overlapping intervals refer to treatment differences with p≤5% (see Materials and methods for calculations of adjustments). Underestimators hold beliefs below and overestimators above reported claims in their group at respective periods.

Figure 1 shows the main results. The upper panels (A–C) show the trend in mean payment claims. The lower panels (D–F) display the number of reported fives over the four payment periods. The left column (panels A and D) shows the general trend for all types subdivided into info (solid black line), control belief (dotted red line) and the control base treatment (dashed green line). The x-axes in the upper panels depict the treatment averages of subjects’ average reported payment claims per round. The “honesty threshold” of 2.5 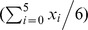 is denoted by the dashed horizontal black lines. The x-axes in the lower panels depict the treatment averages of subjects’ numbers of reported fives per round. The five was taken, because this is the highest possible payout per cast. Here, the “honesty threshold” of two (12⋅1/6) is also denoted by a dashed horizontal black line.

First of all, there is substantial lying in all three treatments. This demonstrates the usefulness of the dice experiment for studying violations of the honesty norm. With respect to mean claimed payments (panel A), people claim on average about 35% more than what they are entitled to: the average claim is about 3.3 CHF per period compared to the honesty threshold of 2.5 CHF. With respect to the number of claimed fives (panel D), people claim about 230% more than what they are entitled to: on average, there are more than four claimed “fives” compared to the honesty threshold of two. All deviances from honesty are highly significant at the 0.1% level. This is shown by confidence intervals of 99.9% of differences between dice reports and honesty thresholds in Fig. S5 in the appendix.

In a first step, the main effect of information feedback was estimated (panel A and D). This was done by computing the trends in dishonesty over the four periods regardless of beliefs about others’ honesty. This analysis can be regarded as a replication of [4] with more data per subject. In [4] the authors used one dice report per subject before and one after information feedback. The present study uses twelve dice reports before information feedback and three times twelve dice reports after information feedback, i.e., considering three periods with twelve reports each.

Fig. 1A shows the main effect of information feedback for all subjects (i.e., regardless of their beliefs) in terms of reported means and Fig. 1D, in terms of reported fives. It can be seen that subjects lie slightly more in subsequent periods if they are informed about others’ reported payment claims. In the information treatment, subjects claim roughly between 0.10 and 0.14 higher average payouts and between 0.1 and 0.4 more fives compared to the control treatments. However, these differences are statistically not significantly different from both control treatments (Table S2). This means that the main “broken windows effect” in this study for all types of people is weak. Information of others’ transgressions triggers actors to increase their own transgressions only modestly. The direction of the effect is similar as in [4] and can be replicated. Yet, the magnitude of the effect is statistically weaker than in [4]. Other recent lab and online experiments yield a similar result of a small and non-significant increase of cheating in the feedback group as reported in [15].

However, what happens if beliefs about others’ dishonesty are taken into account? Will believers in too little dishonesty of others increase their own dishonesty and will believers in too much reduce it? If this were the case, the main effect would conceal the underlying dynamics in panels A and D because it works in the opposite direction for under- and overestimators of others’ dishonesty. Therefore, in a second step, the trends in dishonesty were calculated separately for those believing in too little and those in too much dishonesty of others. Since beliefs were not measured in the control base treatment, trends are only separated between control belief and info treatments.

Panels B, C, E and F of Fig. 1 show the results when types are differentiated by under- and overestimators. It can be seen that the results change substantially, confirming the interaction effect very clearly. “Underestimators” are defined such that they hold beliefs below and “overestimators” above reported payment claims in their group at respective periods. Per round with twelve dice casts, underestimators in periods after information feedback report 0.44 CHF more in average payments than underestimators in the control belief treatment (Fig. 1B, Table 1A). With respect to the number of reported fives, underestimators in the info treatment report 1.3 more fives than underestimators in the control belief treatment (Fig. 1E, Tab. 1B). Both interactions are highly significant at the 0.1% level.

**Table 1:** Linear regression models of treatment differences between info and belief control treatments.

	(A)	(B)
	means	fives
info	−0.715***	−3.454**
	(−3.72)	(−3.30)
underestimator types	−1.114***	−4.362***
	(−6.39)	(−4.51)
info × underestimator types	1.156***	4.741***
	(5.19)	(4.31)
intercept	4.118***	7.630***
	(25.47)	(8.18)
N	480	480

Model A shows differences in claimed mean payments and model B differences in claimed number of fives with respect to under- and overestimators and their treatment interactions. One case refers to the reported mean (model A) or reported number of fives (model B) over the sequence of twelve dice casts per period per subject (yielding a total of N = 480 cases for each model). Only periods 2, 3 and 4 are used because these are the periods after information feedback in the info treatment. Robust standard errors are used, which were clustered for subjects. T statistics are reported in parentheses, stars denote statistical significance with *p<0.05, **p<0.01, ***p<0.001.

The same interaction holds for overestimators. Actors overestimating the extent of lying subsequently adjust their reports downwards compared to the control belief treatment. Per round with twelve dice casts, overestimators in periods after information feedback claim 0.72 CHF less in average payments than overestimators in the control belief treatment (Fig. 1C, Table 1A). With respect to the number of reported fives, overestimators claim 3.5 fewer fives in the info condition compared to overestimators in the control belief condition (Fig. 1E, Table 1B). Both reductions are statistically significant at the 0.1% level. The differences between info and control belief treatments for over- and underestimators are substantial, taking into account that honest subjects would report an average of 2.5 Swiss Francs per period and two occurrences of the highest payout “five”.

Note that the discussed percentage comparisons refer to the average effects over periods 2 to 4, where information feedback was given in the info treatment and withheld in the control belief treatment. The reported percentages in the text can be computed from Table 1. This is done by referring to the intercept as baseline, which represents the mean claimed payments (model A) or average number of reported fives (model B) for overestimators in the control belief treatment. The percentages for the other three groups can be calculated by taking the differences from the baseline with respect to main and interaction effects.

Another way of quantifying the strength of the interaction is to compare the proportion of liars in different treatments. One way of doing this is to compute the expected proportion of the highest payoff five of a fair die (which is 1/6), compare it to the empirically reported proportion of fives π and adjust it for liars who actually threw a five but would have lied in case of lower cast numbers (i.e., multiply by 6/5). This yields an estimate of the proportion of liars λ = (π−1/6)⋅6/5 (see also [13]). The proportion of reported fives for over- and underestimators in the information and control belief treatments can be calculated from Table 1.

The proportion of liars in the population of underestimators is more than twice as large in the info as in the control treatment: there are 25.6% underestimating liars in the info and 12.7% underestimating liars in the control treatment. Note that these percentages refer again to periods 2 to 4, demonstrating that underestimators increase their lying if they are informed about the extent of lying in their group. Moreover, the proportion of liars in the population of overestimators decreases by less than half if they are informed about the extent of liars in their group. There are 56.3% overestimating liars in the control condition and 21.8% overestimating liars in the info treatment.

This shows that lying can either be more than halved or more than doubled depending on subjective beliefs and whether information feedback is provided. This clearly demonstrates that the observed interaction is substantial and gives rise to the conclusion that the direction of the dynamics towards either decay or stabilization of social order is strongly contingent on actors’ subjective beliefs regarding the extent of norm violations in the population. Furthermore, it can be noted that the dynamics become stronger over time in the sense that treatment differences increase over time (Fig. 1), giving additional weight to the conclusion.

## Discussion

The reported results put recent findings about normative dynamics into perspective. Information about norm violations of others does not per se trigger subsequently more norm violations. The mechanism is contingent on actors’ subjective beliefs. Since some norm violations are visible and others go undetected, the dynamics depends on whether actors under- or overestimate the complete rate of norm violations.

If most subjects begin by underestimating transgression of others, it is likely to be a sign that they are normatively oriented in the beginning and project this normative orientation onto others. If they do not hear otherwise, they stick to this belief. Nonetheless, if they hear that they underestimate, they will adjust upward, norm violations will spread in the system, they may become less normatively oriented and adjust subsequently, triggering normative decay up to a society mainly consisting of liars. However, the reversed process is likely for subjects who overestimate from the beginning. They are likely to project that others are also not normatively oriented and stick to that if they do not hear otherwise. If they are informed, and as a result hear otherwise, they scale down their beliefs about cheating to a substantial degree. As a consequence, the honesty norm gains support, and honesty may spread up to a society mainly consisting of moralists.

The experimental findings discard the alternative explanation that the norm-stabilizing effect of underestimating transgressions is solely due to a sanctioning mechanism. It has been argued that this effect occurs because information about others’ norm violations serves as clue to the likelihood or severity of sanctions to be expected [3]. In this line of reasoning, underestimators adjust their beliefs about the probability or severity of sanctions after having learnt about the true prevalence of norm violations. Consequently, underestimators interpret others’ norm violations as an indication that sanctions are less likely or less severe than they originally thought – and increase their own transgressions. This mechanism explains the effect of information about others’ transgressions on increased own transgressions as a selfish, forward-looking reaction of underestimators on their updated beliefs about sanctions. Yet, in the experimental design sanctions have been completely excluded because individual norm violations have not been observable by the experimenters. Therefore, it can be concluded that there is a ‘pure’ effect of the spread of norm violations that is not generated by a change in the perception of sanctions.

The presented interaction between actions and beliefs for the case of lying also provides novel insights for the basic debate about their general interrelation. There is a lively literature on the temporal order of the two – do beliefs determine behavior or does behavior determine beliefs? The first line of reasoning is often advocated by economists and may be called reaction theory, the second one, advocated by psychologists, projection theory [16]. In social dilemmas, reaction theory refers to conditional cooperation. In this line of reasoning, cooperative behavior is a reaction on the actor’s belief that at least a certain fraction of the population will cooperate. It has been shown, for example, that a substantial proportion of actors condition their degree of cooperativeness on their belief that enough others also cooperate [17] and discontinue cooperation if they are informed about a critical fraction of freeriders [18]. Projection theory argues for a reversed causal path: actors project their own cooperative (or non-cooperative) intentions onto others and expect them to be similar to themselves [19], [20]. In this way, cooperative intentions trigger an alignment between cooperative behavior and cooperative beliefs so that beliefs may rather be the consequence of post-hoc rationalizations of behavior than its origin [21].

The data suggests a combination of reaction and projection mechanisms. Subjects without information feedback show a constant lying pattern, which is strongly correlated with their beliefs. Underestimators start with comparably few lies and seem to project their lying behavior onto the lying behavior of others since they retain their honesty level throughout the game. Conversely, overestimators start with comparably many lies and also seem to project their lying behavior onto others, retaining their dishonesty level. The dynamics in the treatment with information feedback, however, supports reaction theory. Informed underestimators react on their updated belief and adjust their lying behavior upwards. Similarly, informed overestimators react on their updated belief and adjust downwards. This combined mechanism of projection and reaction has also been supported in a recent study on cooperation in an asymmetric social dilemma [22] and it is likely to have played a role in field experiments on broken windows [1], [5].

Furthermore, the study sheds a novel light on the debate about peer effects in social dilemmas. It is often argued that peer effects are biased towards people’s self-interest, triggering asymmetric peer effects in social dilemmas. For example, people often exhibit a self-serving bias in their perception of their own cooperativeness [23] and are more averse towards envy than guilt [24]. In a novel study, the possible confounding between peer effects and strategic incentives in earlier studies has been ruled out by design [22]. The authors show in the context of a gift-exchange game that agents revise their level of reciprocity between the principal’s offered wage and their returned effort contingent on information about another unrelated agent’s effort. These peer effects are shown to be strongly asymmetric: Agents only make downward adjustments which are in their own self-interest and do not increase their effort if the other agent has shown higher effort compared to their own. In contrast, my study does not reveal such an asymmetry with predominantly self-interested adjustments. Honesty revisions occur in both directions – actors exhibit more but also less lying after information feedback, based on whether they under- or overestimated the extent of lying in the population.

One may reason whether the above discussed symmetry of self-serving and self-harming honesty adjustments may be caused by the monetary incentives for correct beliefs. There is evidence that the measurement of incentivized beliefs can have consequences for the measurement of cooperative behavior. If people receive money for accurate beliefs, they behave more cooperatively compared to setups without such premiums [25]. One explanation for this may be that subjects aim for a specific minimum payment as their final earnings for participating in the experiment. Hence, subjects without monetary compensation for accurate beliefs may compensate this relative “mental” loss by more freeriding in order to increase their earnings. However, if this were true for my setup, compared to a version without incentivizing beliefs, there would be higher honesty levels for both kinds of subjects, over- and underestimators. Therefore, this argument does not explain why overestimators decrease their lying about as much as underestimators increase theirs. In addition, it has been shown that monetary incentives for beliefs increase belief accuracy [25]. A likely reason is that subjects take belief elicitations more seriously when getting paid for accuracy. Hence, incentivized beliefs yield more accurate belief measures and thus have more predictive power. This gives more weight to the conclusion that information feedback in my study yields behavioral adjustments in both directions – more and less honesty. Subsequent studies should explore this issue in more detail and also investigate whether the kind of dilemma may explain the differences between both studies.

The group sizes of under- and overestimators in the analysis deserve some further discussion. The fraction of under- and overestimators varies over time and experimental conditions; however, it is always sufficiently large for robust estimations. Over all, there are more under- than overestimators, and the fraction of underestimators varies between 44% and 89%. Initially, most subjects underestimate the extent of norm violations. With respect to reported means, there are 70% underestimators in the info and 73% in the control belief condition. Similarly, there are 83% initial underestimators of reported fives in both conditions. Over time, the fraction of underestimators decreases in the info condition, whereas it remains at a roughly constant level between 72% and 84% in the belief control condition. Detailed time trends of the fraction of under- and overestimators and respective confidence intervals are given in the (Fig. S6).

The causal interpretation of the differential effects for under- and overestimators deserves some caution, however. It has to be noted that the groups are determined by subjects’ behaviors during the course of the lying experiment and not via random assignment. Hence, it cannot be ruled out that effects of third, unmeasured variables may mitigate some of the observed dynamics. Nevertheless, it seems hardly feasible to use random assignment instead since it is not possible to assign subjective beliefs externally. Furthermore, it can be doubted that such strong and straightforward interactions as presented here may be completely driven by other, unmeasured mechanisms.

In summary, the findings show that it is important to take subjective beliefs about undetected norm violations of others into account. Such beliefs can serve as strong mediators for disorder effects on norm conformity. The projection of own norm violations on others and their offsetting dynamics into both normative decay and norm conformity with respect to beliefs deserves further study.

## Materials and Methods

### Experimental Design

The experiment was conducted as follows: Subjects were recruited from the address pool of the DeSciL laboratory at ETH Zurich, consisting of students from all disciplines from ETH and the University of Zurich. Subjects were invited in groups of ten and received 5 CHF show-up fee. The experiment consisted of 24 sessions with ten subjects per session. Subjects were first informed about the general instructions of the experiment before the actual experiment started (see Fig. S1 for paper instructions and exact wording). Each subject was assigned an isolated cubicle where nobody could observe subjects’ actions. All subjects started after all were finished reading the paper instructions. All subjects completed each round of dice casts in parallel.

In each session, each of the ten subjects per group was randomly allocated to one of three treatments, yielding an allocation of 3-3-4 in each treatment, with random variations of group sizes per treatment in each session. It was ensured that there was an equal division of 80 subjects per treatment over all sessions.

In each round, each subjects had to report twelve dice casts using a computer interface (Fig. S2 displays exact wordings and graphical animations). Note that subjects were allowed to perform additional casts to verify that the die was working properly. It was pointed out that only the first twelve casts “count” as payments. This procedure was implemented to enhance subjects’ trust in not being deceived by the experimenter and to generate higher levels of lying and with it more explanatory power due to larger variance. The latter argument is based on previous dice experiments, where subjects were more comfortable to report “delayed” higher casts than invented higher casts [26].

There was one trial period without payments and four rounds of dice casts with payments. In the payment-relevant rounds, one reported dice cast was randomly selected for payouts. This was common knowledge (see paper instructions Fig. S1).

Subjects were asked about their beliefs in the info and in the control belief treatments in the following way (Fig. S3 shows exact wordings and graphical animations). Subjects had to estimate the number of reported payoffs for each reported dot. Thus, six numbers were elicited from each subject: the number of reported payoffs of zero, one, two, three, four, and five. The elicitation of beliefs was incentive compatible using a similar logic to the quadratic scoring rule [27]. The difference between each of the six estimated payoff reports and the actual number in the session was computed. A perfect guess yielded a payoff of 0.80 Swiss Francs (CHF), an absolute difference of 1 yielded 0.75 CHF, an absolute difference of 2 yielded 0.60 CHF, a difference of 3 yielded 0.35 CHF and a difference equal or larger than 4 yielded zero CHF (see Table S2).

The logic behind this is that each squared deviation is multiplied with 5 and subtracted from the best payoff of 0.80 CHF. The payoff π is computed as 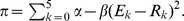 Here the best payoff is α. This payoff is reduced with differences between the estimate E_k_ for the reported dot *k* and the real value R_k._ This difference is scaled with β. Note that only positive payoffs were paid out and negative ones truncated and transformed to zero. In the experiment, α = 80 and β = 5, yielding the payouts from Table S1.

In the information treatment, subjects were informed about the number of reported payoffs for each reported dot of the nine other subjects in their group (see Fig. S4 for exact wording and graphical animations). They were informed about their own estimates and these were compared with the actual frequencies. In the control belief treatment, subjects also estimated the number of reported payoffs of the other participants of their group. Also in the control belief treatment, subjects received money for belief accuracy at the end of the experiment; however, they did not receive information feedback. There was no measurement of beliefs and no information feedback in the control base treatment.

### Duration and Average Payouts of the Experiment

The experimental sessions lasted twenty-four minutes on average. Subjects received on average 26 CHF, consisting of 21 CHF for payouts from decisions and 5 CHF show-up fee. The payoffs ranged between 15 and 42 CHF. More specifically, average payments for dice casts were 13 CHF, ranging from 4 to 20 CHF. Beliefs yielded 6 CHF on average, ranging from 0 to 12 CHF.

### Calculation of Adjusted Confidence Intervals

Error bars in Figure 1 show adjusted 95% confidence intervals such that non-overlapping intervals refer to treatment differences with p≤5%. The reason for the adjustment is that the Figure is constructed such that non-overlapping confidence intervals refer to hypotheses tests with an error rate of 5%. Since the error rate of two non-overlapping standard 95% confidence intervals is smaller than 5%, yielding a too conservative test, confidence intervals are adjusted in order to represent hypothesis tests of differences in means at a 5% error rate [28], [29].

The confidence intervals are adjusted such that the type I error rate when comparing the overlap of 100(1-γ)% is 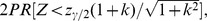 with k as the ratio of standard errors, Z as the normal variate and γ as the value of the adjusted confidence interval. For example, if the standard errors for two means are equal (k = 1), this yields γ = 0.166, meaning that non-overlapping 83% confidence intervals represent significantly different means with a 5% error rate. The larger the ratio of standard errors, the larger the adjusted confidence level (e.g., a ratio of k = 5 yields adjusted 90% confidence intervals referring to a 5% error rate).

For Figure 1, the ratios of standard errors of the means in the info and the control belief treatments were computed and respective adjusted confidence levels were calculated using self-developed Stata code. Note that in Panels A and D, with three comparisons of means, the largest ratio of standard errors was used. The calculated standard errors in Figure 1 range from 84.0% to 86.0% confidence intervals, which all reflect hypothesis tests with a 5% error rate.

### Ethics Statement

Participants of the laboratory experiment were recruited from the address pool of the ETHZ Decision Science Laboratory at ETH Zurich (hereafter DeSciL). The address pool consists of students from all disciplines from ETH and the University of Zurich. The experiment fully adhered to the Operational Rules of DeSciL. The Operational Rules are public and are published online at the link given in reference [30]. Subjects were recruited by e-mail and were informed that the experiment would take place at DeSciL and hence adhered to the Operational Rules of DeSciL.

The experiment adhered to the rule of no deception, a rule that is standard in experimental economics and is becoming standard in experimental sociology. This means that subjects were in no way deceived or lied to, and all instructions truthfully described all procedures of the experiment. This is stated clearly in the Operational Rules (p. 3): “Deception of research participants is strictly forbidden in the DeSciL. Under no circumstances will participants be lied to or deceived by researchers in any way, before, during, or after the course of a research session. The DeSciL is very concerned about developing and maintaining an unblemished reputation among the research population for transparency and perfect honesty. This rule of no deception applies to the recruitment process, the instructions provided during the experimental sessions, the experimental process and feedback during the research, and the economic compensation participants receive for taking part in the experiment”.

Before the experiment, participants were informed about the monetary compensation for their participation and performance in the experiment. This reflects the Operational Rules as follows (p. 4): “All research participants will be compensated for their time and efforts when taking part in research. A show up payment is guaranteed to all participants who sign up for a particular study and show up to the laboratory on time”. Monetary incentives were used to ensure thoughtful and careful decisions (p. 4): “Experiments should be conducted in such a way that behavior is incentive compatible. Careful and thoughtful decision making should be encouraged and remunerated”.

Subjects participating in experiments in DeSciL have the right of voluntary participation (p.4):“ Every laboratory participant has the right to terminate their involvement in research at any time for any reason. If a research participant chooses to exercise this right, they are still entitled to receive their show-up payment, although additional potential earnings are relinquished. There shall be no penalty levied against a participant who chooses to terminate their research participation”.

It is optional in DeSciL to provide written or verbal informed consent. This is stated in the Operational Rules as follows (p.4):“It is optional that research participants complete a consent form before participating in a research session. A typical consent form contains: the name of the research session, including the time and date; verification that the participant is in the laboratory of their own volition; a claim of understanding of the basic rights as a research participant, including the right to terminate research participation at any time for any reason; contact information for the responsible researchers should the participant have follow up questions or concerns”. Since all procedures adhered to the Operational Rules of DeSciL and since these are publicly available, it was deemed unnecessary that participants complete a consent form before participating in the experiment. The Operational Rules include all necessary information such as no deception, incentive compatible decisions, and participants’ rights to terminate the experiment at any time.

Anonymity of subjects’ decisions and confidentiality was guaranteed and is stated in the Operational Rules as follows (p.4 f.): “The confidentiality of participants will be guaranteed before the research session, during the research activities, during the payment process, and after the completion of the research activities”.

The review committee of DeSciL is called “Review Board”, whose members are listed on the DeSciL website (http://www.descil.ethz.ch/people). All experiments in DeSciL are under the supervision of the Review Board. The Review Board granted me a waiver to perform this research without specific ethical approval.

All information provided during the experiment is given in the Supporting Information. All subjects received paper instructions in German about the procedures and payments at their individual booths. The English translation of these instructions is given in Figure S1. Figures S2–S4 show screenshots of the computer interface of all experimental treatments, including written instructions and input windows.

The research reported in this paper took place exclusively in Switzerland, the country of my university affiliations. All the research was performed in the DeSciL laboratory at ETH Zurich, Haldeneggsteig 4, CH-8092 Zurich.

### Supporting Information

Figures S1–S6 and Tables S1–S2, including paper instructions in all experimental treatments, screenshots of computer interfaces of dice reports, belief elicitations and information feedback, trends of reported payment claims in means and fives with 99.9% confidence intervals, groups sizes of under- and overestimators, payments for belief accuracy and linear regression models of treatment differences in reported means and fives.

Data is available on request from the corresponding author.

## Supporting Information

Figure S1 **Paper instructions in all experimental treatments (English translation).**
(PDF)

Figure S2 **Computer interface for elicitation of payment claims in all treatments.** (Instructions are translated into English, text with arrows give translations for parts of the computer screen).
(PDF)

Figure S3 **Computer interface for belief elicitation in info and belief control treatments.** (Instructions are translated into English, text with arrows give translations for parts of the computer screen).
(PDF)

Figure S4 **Computer interface of information feedback in info treatment.** (Instructions are translated into English, text with arrows give translations for parts of the computer screen).
(PDF)

Figure S5 **Trend of reported payment claims in means (panel A) and fives (panel B) with 99.9% error bars.** All error bars do not overlap with respective honesty thresholds, showing highly significant lying in all treatments at all periods.
(PDF)

Figure S6 **Group sizes of under- and overestimators over periods.** Panel A displays the fraction of underestimators of reported means and panel B, of reported fives. Error bars show adjusted 95% confidence intervals such that non-overlapping intervals refer to treatment differences with p≤5% (see the section Materials and Methods for calculations of adjustments). Underestimators hold beliefs below reported payment claims in their group at respective periods.
(PDF)

Table S1 **Payoffs for accuracy in beliefs in treatment 1 in Swiss Rappen (1 CHF = 100 Rappen).**
(PDF)

Table S2 **Linear regression models of treatment differences in reported means (models 1–2) and fives (models 3–4), referring to average effects for all types.** Models 1 and 3 show differences between info and control belief treatments and models 2 and 4 between info and control base treatments. Only periods 2, 3 and 4 are used, because these are the periods after information feedback in the info treatment.
(PDF)
