# Supplementary material for: Beliefs about Lying and Spreading of Dishonesty: Undetected Lies and Their Constructive and Destructive Social Dynamics in Dice Experiments
Source: PLoS One. 2013 Nov 13;8(11):e77878. doi: 10.1371/journal.pone.0077878 (PMC3827202; doi:10.1371/journal.pone.0077878)
Supplement: Figure S1 — Paper instructions in all experimental treatments (English translation). (PDF) [file pone.0077878.s001.pdf]

Institute of Sociology, University of Zurich

ETH
Eidgenössische Technische Hochschule Zürich
Swiss Federal Institute of Technology Zurich
D-Gess | Chair of Sociology

Procedure of the study

You find a die at your place. In what follows, you are asked to **cast the die 12 times** and to fill in all of your scored points into the computer interface**.** Your entries are completely **anonymous**. Your die casts can neither be observed by other participants nor by the experimenter. Therefore, it is neither possible during the experiment, nor when payments are cashed nor in retrospect to determine your actual die casts or actual die casts of other participants.

Your payments in Swiss Francs (CHF) are calculated according to the table below.

Casted number	6	1	2	3	4	5
Payment in CHF	0.00	1.00	2.00	3.00	4.00	5.00

Please notice that the casted number "six" yields no payment. As you will have realized, the average payment for a large number of die casts is 2.5 CHF per cast (0+1+2+3+4+5)/6 = 2.5 CHF.

One of your die casts is selected randomly for payments in cash. The other 11 die casts are not paid out. During the study, you will run through several rounds; in all of which one die cast is paid out in cash.

Please do not start until you are called upon. After you have finished your required twelve die casts, you are allowed to cast additional casts to verify that the die is working properly. However, only your first 12 casts count.

During the complete study, you are member of a **group of 10 participants altogether**. All the other 9 participants have received the same instructions as you did and are also required to cast 12 times. For everybody, one die cast is randomly selected for payment in cash.

You will run through one trial round, in which you can familiarize yourself with the procedure of the experiment. The trial round has absolutely no effect on your payments. All 10 participants of your group run through the trial round. The main study starts after you and all other members of your group have concluded the trial round. In the main study, your entries are relevant for payments.

Please press the **continue** button as soon as you have finished reading and have no further questions. If you have questions, you can raise your hand. We will come to your place and answer your questions. The experiment starts as soon as all members of your group have pressed the continue button.
