# Supplementary material for: Beliefs about Lying and Spreading of Dishonesty: Undetected Lies and Their Constructive and Destructive Social Dynamics in Dice Experiments
Source: PLoS One. 2013 Nov 13;8(11):e77878. doi: 10.1371/journal.pone.0077878 (PMC3827202; doi:10.1371/journal.pone.0077878)
Supplement: Figure S2 — Computer interface for elicitation of payment claims in all treatments. (Instructions are translated into English, text with arrows give translations for parts of the computer screen). (PDF) [file pone.0077878.s002.pdf]

This is the start of the main study. From now on, your entries are payment relevant. One of your die casts will be randomly selected for payments in cash.

Please cast 12 times your die and fill in your scored points into the following table.

casted number →	Augenzahl	6	1	2	3	4	5
payment →	Auszahlung	0 CHF	1 CHF	2 CHF	3 CHF	4 CHF	5 CHF
cast 1 →	Wurf 1	●	○	○	○	○	○
cast 2 →	Wurf 2	●	○	○	○	○	○
cast 3 →	Wurf 3	○	○	○	○	●	○
cast 4 →	Wurf 4	○	○	○	●	○	○
cast 5 →	Wurf 5	○	○	○	○	○	●
cast 6 →	Wurf 6	○	●	○	○	○	○
cast 7 →	Wurf 7	○	○	●	○	○	○
cast 8 →	Wurf 8	●	○	○	○	○	○
cast 9 →	Wurf 9	○	○	○	●	○	○
cast 10 →	Wurf 10	○	○	○	○	○	●
cast 11 →	Wurf 11	○	○	○	○	○	●
cast 12 →	Wurf 12	○	○	○	○	○	○

continue → Weiter
