# Supplementary material for: Beliefs about Lying and Spreading of Dishonesty: Undetected Lies and Their Constructive and Destructive Social Dynamics in Dice Experiments
Source: PLoS One. 2013 Nov 13;8(11):e77878. doi: 10.1371/journal.pone.0077878 (PMC3827202; doi:10.1371/journal.pone.0077878)
Supplement: Figure S3 — Computer interface for belief elicitation in info and belief control treatments. (Instructions are translated into English, text with arrows give translations for parts of the computer screen). (PDF) [file pone.0077878.s003.pdf]

Please estimate the reported payments of the other 9 participants of your group. For this purpose, enter the frequencies of claimed payments in the table below. Take into consideration that you have to estimate the distribution of a total of 108 die casts: because each of the 9 participants report 12 die casts, the total number of reported die casts is 9 * 12 = 108.
As you will have realized, the average frequency for each reported payment claim is 18 for a large number of die casts: 1/6 x 12 casts x 9 participants = 18.

You receive additional money for the accuracy of your estimates: the better your estimates, the more money. You are paid separately for each of your 6 estimates, that is, you are paid for your estimate of the frequency of reported 0 CHF, 1 CHF, 2 CHF, 3 CHF, 4 CHF and 5 CHF. You are paid 0.80 CHF for a correct value. You are paid 0.75 CHF for a deviation of 1 from the correct value. For a deviation of 2, you are paid 0.60 CHF, for a deviation of 3, you are paid 0.35 CHF and you are paid nothing for larger deviations.

reported cast → frequency →

Auszahlung	Häufigkeit
0 CHF	17
1 CHF	13
2 CHF	22
3 CHF	19
4 CHF	26
5 CHF	11
total number →	Gesamtzahl: 108

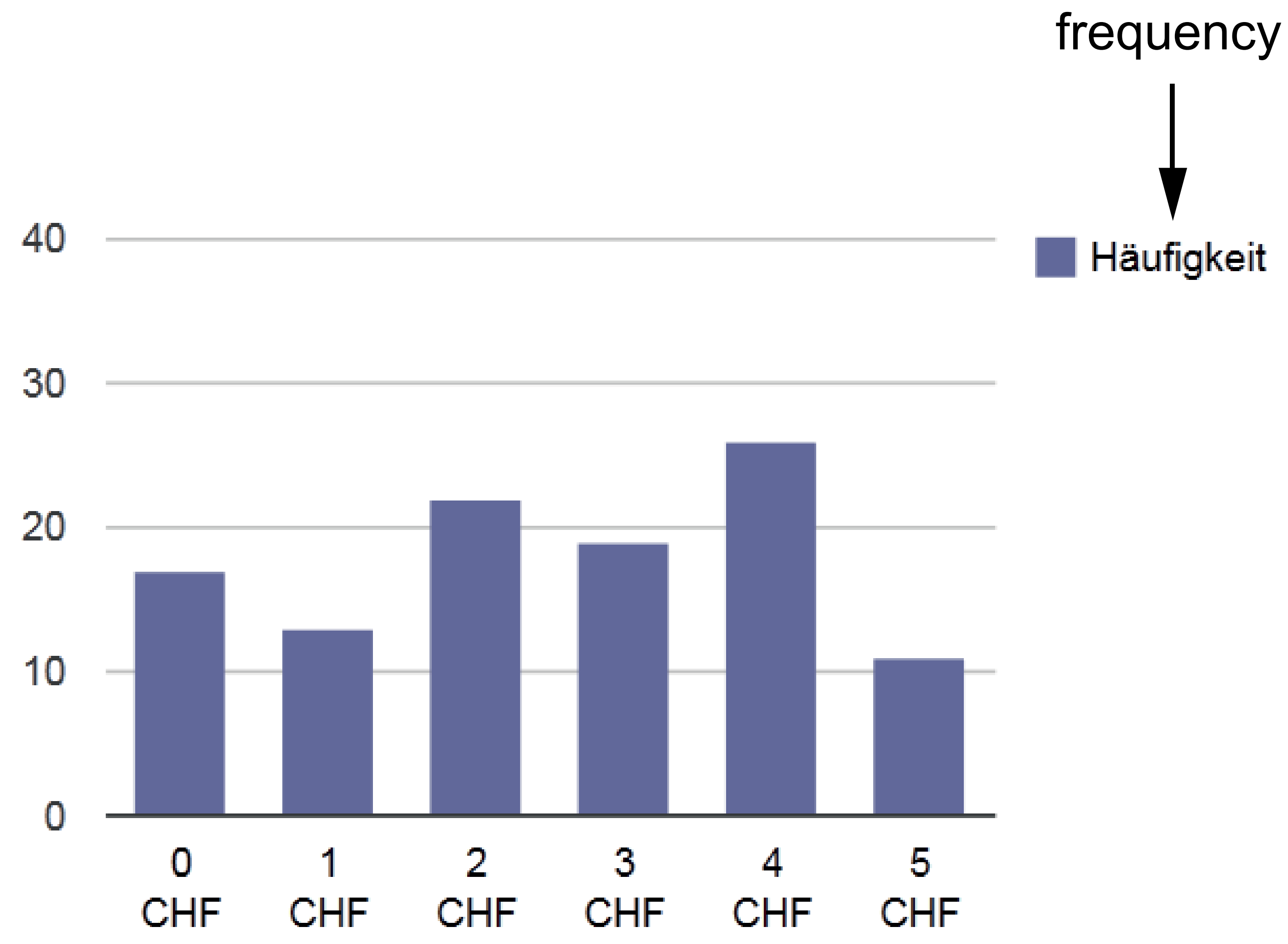

Weiter ← continue
