# Supplementary material for: Beliefs about Lying and Spreading of Dishonesty: Undetected Lies and Their Constructive and Destructive Social Dynamics in Dice Experiments
Source: PLoS One. 2013 Nov 13;8(11):e77878. doi: 10.1371/journal.pone.0077878 (PMC3827202; doi:10.1371/journal.pone.0077878)
Supplement: Figure S4 — Computer interface of information feedback in info treatment. (Instructions are translated into English, text with arrows give translations for parts of the computer screen). (PDF) [file pone.0077878.s004.pdf]

The following figure shows your estimates in comparison to the actual values.

frequency
your estimates

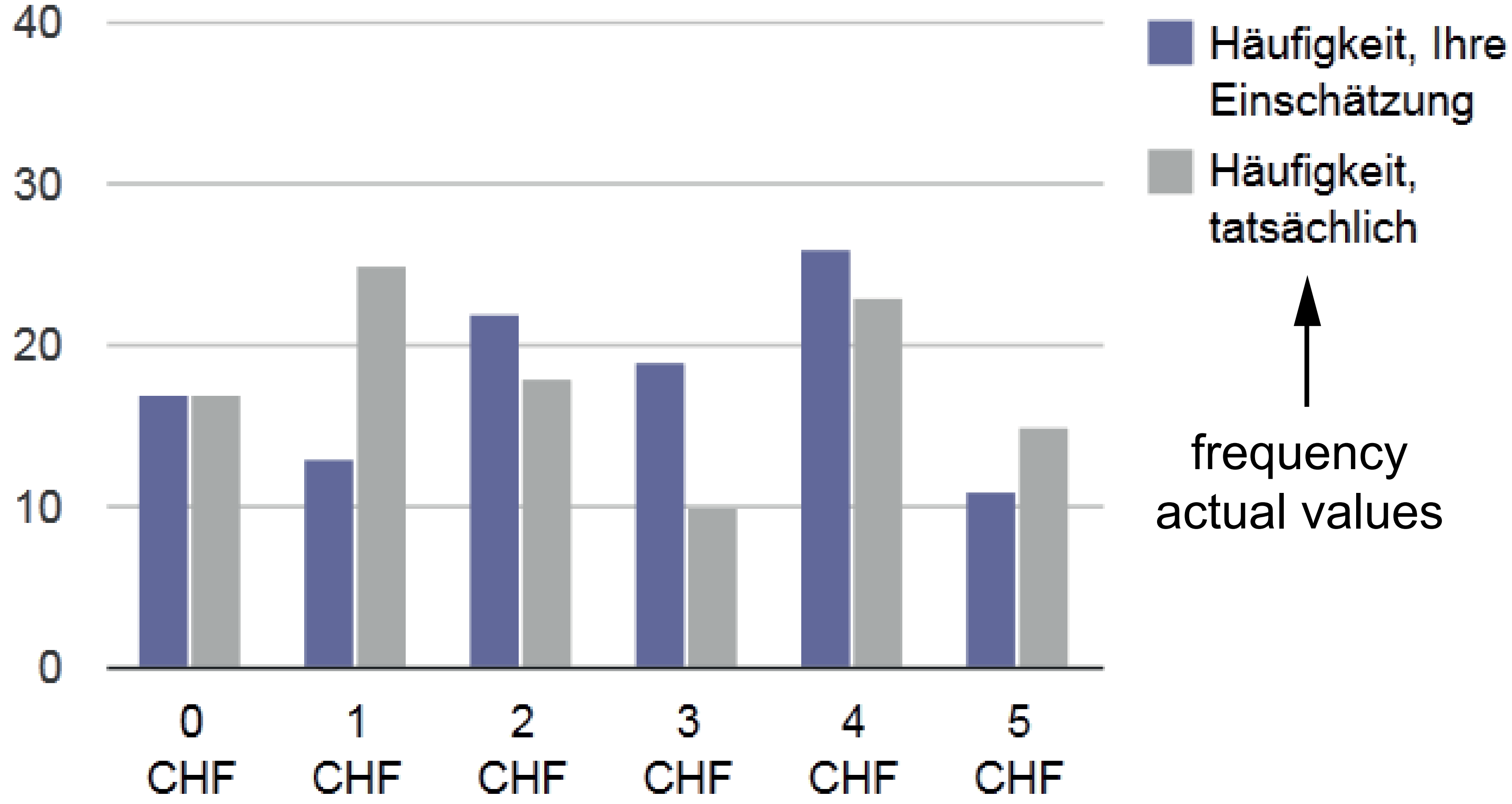

frequency
actual values

Für Ihre Einschätzung erhalten Sie 1.15 CHF.

Your receive 1.15 CHF for your estimates.

Weiter

continue
