# Supplementary figures and images for: Beliefs about Lying and Spreading of Dishonesty: Undetected Lies and Their Constructive and Destructive Social Dynamics in Dice Experiments
Source: PLoS One. 2013 Nov 13;8(11):e77878. doi: 10.1371/journal.pone.0077878 (PMC3827202; doi:10.1371/journal.pone.0077878)

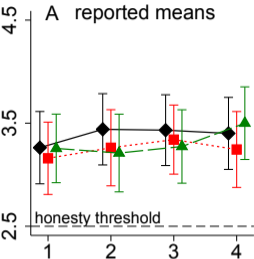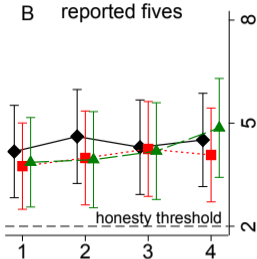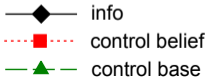

Supplement: Figure S5 — Trend of reported payment claims in means (panel A) and fives (panel B) with 99.9% error bars. All error bars do not overlap with respective honesty thresholds, showing highly significant lying in all treatments at all periods. (PDF) [file pone.0077878.s005.pdf]

A reported means

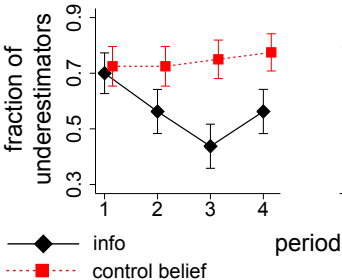

B reported fives

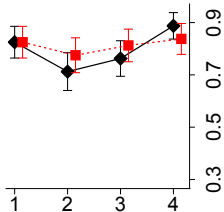

Supplement: Figure S6 — Group sizes of under- and overestimators over periods. Panel A displays the fraction of underestimators of reported means and panel B, of reported fives. Error bars show adjusted 95% confidence intervals such that non-overlapping intervals refer to treatment differences with p≤5% (see the section Materials and Methods for calculations of adjustments). Underestimators hold beliefs below reported payment claims in their group at respective periods. (PDF) [file pone.0077878.s006.pdf]
