# Supplementary material for: Beliefs about Lying and Spreading of Dishonesty: Undetected Lies and Their Constructive and Destructive Social Dynamics in Dice Experiments
Source: PLoS One. 2013 Nov 13;8(11):e77878. doi: 10.1371/journal.pone.0077878 (PMC3827202; doi:10.1371/journal.pone.0077878)
Supplement: Table S1 — Payoffs for accuracy in beliefs in treatment 1 in Swiss Rappen (1 CHF = 100 Rappen). (PDF) [file pone.0077878.s007.pdf]

Difference belief and real frequency of reported payoff	0	1	2	3	≥ 4
CHF	0.80	0.75	0.60	0.35	0
