# Supplementary material for: Beliefs about Lying and Spreading of Dishonesty: Undetected Lies and Their Constructive and Destructive Social Dynamics in Dice Experiments
Source: PLoS One. 2013 Nov 13;8(11):e77878. doi: 10.1371/journal.pone.0077878 (PMC3827202; doi:10.1371/journal.pone.0077878)
Supplement: Table S2 — Linear regression models of treatment differences in reported means (models 1–2) and fives (models 3–4), referring to average effects for all types. Models 1 and 3 show differences between info and control belief treatments and models 2 and 4 between info and control base treatments. Only periods 2, 3 and 4 are used, because these are the periods after information feedback in the info treatment. (PDF) [file pone.0077878.s008.pdf]

	(1) mean (control belief)	(2) mean (control base)	(3) fives (control belief)	(4) fives (control base)
info	0.142	0.0965	0.371	0.146
	(1.06)	(0.71)	(0.69)	(0.27)
intercept	3.282***	3.328***	4.104***	4.329***
	(34.08)	(33.79)	(10.85)	(11.33)
N	480	480	480	480

t statistics in parentheses, robust s.e. clustered for subjects, * $p < 0.05$, ** $p < 0.01$, *** $p < 0.001$
